# Biomechanical Characterization of Cardiomyocyte Using PDMS Pillar with Microgrooves

**DOI:** 10.3390/s16081258

**Published:** 2016-08-09

**Authors:** Nomin-Erdene Oyunbaatar, Deok-Hyu Lee, Swati J. Patil, Eung-Sam Kim, Dong-Weon Lee

**Affiliations:** 1 MEMS and Nanotechnology Laboratory, Mechanical Engineering, Chonnam National University, Gwangju 61186, Korea; nominerdene16@yahoo.com (N.-E.O.); cinsea@naver.com (D.-H.L.); swatipatil39@gmail.com (S.J.P.); 2 Department of Biological Sciences, Chonnam National University, Gwangju 61186, Korea; eungsam.kim@jnu.ac.kr

**Keywords:** polydimethylsiloxane pillar, cardiomyocyte, microgrooves, contraction force, drug screening

## Abstract

This paper describes the surface-patterned polydimethylsiloxane (PDMS) pillar arrays for enhancing cell alignment and contraction force in cardiomyocytes. The PDMS micropillar (μpillar) arrays with microgrooves (μgrooves) were fabricated using a unique micro-mold made using SU-8 double layer processes. The spring constant of the μpillar arrays was experimentally confirmed using atomic force microscopy (AFM). After culturing cardiac cells on the two different types of μpillar arrays, with and without grooves on the top of μpillar, the characteristics of the cardiomyocytes were analyzed using a custom-made image analysis system. The alignment of the cardiomyocytes on the μgrooves of the μpillars was clearly observed using a DAPI staining process. The mechanical force generated by the contraction force of the cardiomyocytes was derived from the displacement of the μpillar arrays. The contraction force of the cardiomyocytes aligned on the μgrooves was 20% higher than that of the μpillar arrays without μgrooves. The experimental results prove that applied geometrical stimulus is an effective method for aligning and improving the contraction force of cardiomyocytes.

## 1. Introduction

The heart is the most important organ and performs a vital function in living organisms; it consists of atria, ventricles, and valves that contract regularly and continuously. Cardiomyocytes arranged in parallel on the ventricle walls periodically contract and relax to circulate blood throughout the body [1]. When the cardiomyocytes contract and relax, they make the chambers bigger and smaller, which pushes blood into the blood vessels and they are again filled with blood coming back into the heart. Analyses of any abnormal contraction or relaxation of the cardiomyocytes are important, as they can enhance understanding of cardiovascular diseases and help to evaluate drug toxicity [2,3,4]. With regard to drug toxicity, bio-hybrid systems represent a new and promising paradigm for engineering, aiming at solving technological issues by means of integration of living biological components [5]. Also, biological research has aimed for qualitative understanding of fundamental phenomena at the molecular and cellular scales through new programs at the intersection of engineering and biology [6].

Electrophysiology-based assays for the interactions of compounds with hERG (the human ether-à-go-go-related gene) channels are mainly used for in vitro methods to screen the cardiac toxicity of drugs [7,8]. Especially, this assay is useful for pharmaceutical industries to recognize drug interaction with ion channels as early as possible in the screening process. This method, called a patch clamp, measures the changes in voltage-gated ions based on the action potential (AP) of cardiomyocytes. However, these assays are only applied to a single cell at a time and are unsuitable for measuring cell mechanical properties such as contraction force [9]. Other issues with hERG assays are that the method for detecting ion channel current change is an inconvenience for high-throughput drug screening and it is a low efficiency procedure because it is highly dependent on the technician’s individual skill and requires a great deal of time. Microelectrode arrays (MEAs) are a technique seeing more use, and they have been shown to yield valuable information on neural network and cardiac tissue dynamics. The production of MEAs is typically trusted to thin film technologies derived from the micro-electronics manufacturing industry, resulting in the monetary value of chips increasing markedly with the size and number of array elements. However, MEAs technology is inconceivable for measuring the mechanical properties of the cells, and another issue is their scalability [10]. Recently, a variety of methods for quantitatively evaluating the contraction force have been developed to better understand the mechanics and physiology of cardiomyocytes, namely, the abnormal characteristics of cardiomyocytes under the effect of drugs [11]. Recently, many 3D structured biocompatible materials and hybrid methods have been developed to better understand the mechanical and electrical stimulation of mammalian and primary cell function. For instance, in healthcare systems in general, biodegradable polymers have become highly important in the field of biomaterials and tissue engineering, due to the avoidable additional surgery to remove the implants or scaffolds [12]. The electrospinning is an efficient technique employed for fabricating polymeric micro- to nanometer scaled fibers. The pattern structure of polymer/drug composite can lead to the development of ultrafine micro and nanometer structures for a host of applications [13].

Microelectromechanical systems (MEMS) devices such as micro-cantilevers have been proposed for measuring the cardiomyocytes’ contraction force [14,15,16]. The contractions and relaxations of cardiomyocytes create mechanical bending in the micro-cantilevers; the degree of bending can be measured using an optical microscope to evaluate the contraction force. However, the chip (thin layer-PDMS) fabrication process is more complicated and is challenging when performing high-throughput drug screening, as applying the force to the substrate will produce a wrinkled pattern and make it impossible to precisely determine the location and directional vector of point forces [17,18]. Also, a different method has been utilized for evaluating the contraction force of cardiomyocytes using the micro-pillar (μpillar) arrays [19,20,21,22]. Tanaka et al. demonstrated that PDMS-based cardiomyocyte bio-micro actuator fabricated using PDMS μpillars driven to repetitive motion by attached pulsating cardiomyocytes. Ribeiro et al. improved force transduction by contractile neoCMs connected to PDMS microposts following covalent bonding of laminin to PDMS surface with organo-silanes. Park et al. cultured cells on micro cantilevers and measured cardiomyocyte contraction force by bending displacement. The μpillar arrays were made using biocompatible and mechanical compliance material like polydimethylsiloxane (PDMS)-silicone elastomer and cardiomyocyte cultivated on the top surface. However, the cardiomyocytes are arranged isotropically, causing less mechanical bending and irregular contraction force, which results in relatively small contractions and less accuracy during drug screening evaluations. 

In the present work, to enhance the contraction force of cardiomyocytes, the tops of μpillar arrays were patterned with microgrooves (μgrooves) causing the cardiomyocytes to contract in a fixed direction. μpillar arrays with µgrooves were manufactured using a micro-molding process with PDMS in a double-layered SU-8 structure. Additionally, MATLAB was used to develop a graphic user interface (GUI)-based image analysis program to quantitatively analyze the cardiomyocytes’ contraction force and direction for potential high-throughput drug screening in the future. 

## 2. Material and Methods

### 2.1. Design and Fabrication of Pillar Arrays without and with Microgrooves

The structure of the heart cell organized on multiple scales helps to promote effective blood ejection from the ventricles and direct the collective movement of action potentials (AP) along the tissue to provide strong synchronous contraction. Before determining the μpillar arrays’ geometry, we consider the studies showing that the alignment of cardiomyocytes was improved on a PDMS surface patterned with μgrooves [23,24,25], while the cardiomyocytes grew anisotropically on an unpatterned surface [26,27]. Cardiac tissue alignment has been studied at all levels, ranging from nano- to micron-scale (3D) with a bio-mimic topographic environment. Daniel Carson et al. [28] reported underlying nanoscale topographical cues were capable of orientation of the cardiac extracellular matrix. Teixeira et al. [29] demonstrated that for groove widths ranging from 950 nm to 330 nm, cell alignments were significantly greater in 600 nm deep groove size compared to the other grooves. Meanwhile, groove size is considered an important factor in cell growth; the cell growth and adhesion is hindered when the µgrooves are too wide and deep. The excessively deep and shallow groove size is an insignificant influence for cell spreading condition; also, too narrow a pitch is unable to provide whole cell adhesion and structural maturation [30]. In Euler-Bernoulli Beam theory, the elastic beam is deflected due to the lateral force which is produced by cardiomyocyte contraction on the micro pillar. To calculate a small amount of cardiomyocyte contraction force at the single cell level, the beam must be highly flexible and elastic. To produce a low spring constant with a highly flexible micro pillar, the aspect ratio of the μpillar is also one of the important parameters.

**Figure 1:**
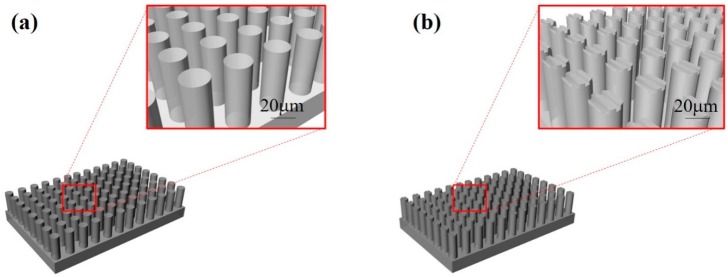
Schematic diagrams of PDMS μpillar arrays (**a**) without and (**b**) with μgrooves.

Therefore, in the present work, µgrooves with a width of 1.5 μm and a depth of 0.5 μm were created on the top of μpillars with a diameter of 16 μm to improve the alignment and adhesion of the cardiomyocytes. Moreover, to prevent the cardiomyocytes from falling down between the μpillar arrays, the space between the μpillars was set as 7 μm with a μpillar diameter of 16 μm and 48 μm in length. In general, the size of a single cardiomyocyte on day 1 is roughly 10 μm and its length will increase up to 100 μm under growth conditions. The schematics of μpillar arrays fabricated with PDMS without and with μgrooves are shown in Figure 1a,b, respectively.

**Figure 2:**
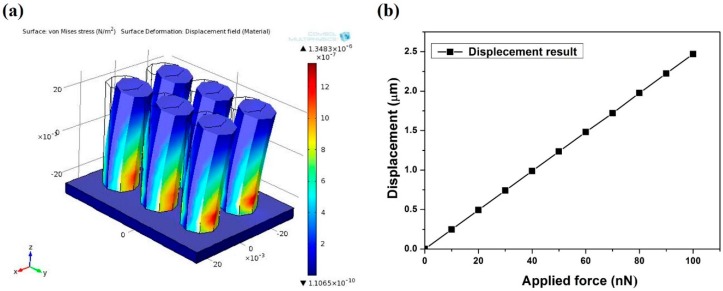
(**a**) Finite element analysis of PDMS μpillar arrays and (**b**) displacement of μpillar as a function of applied force.

**Figure 3:**
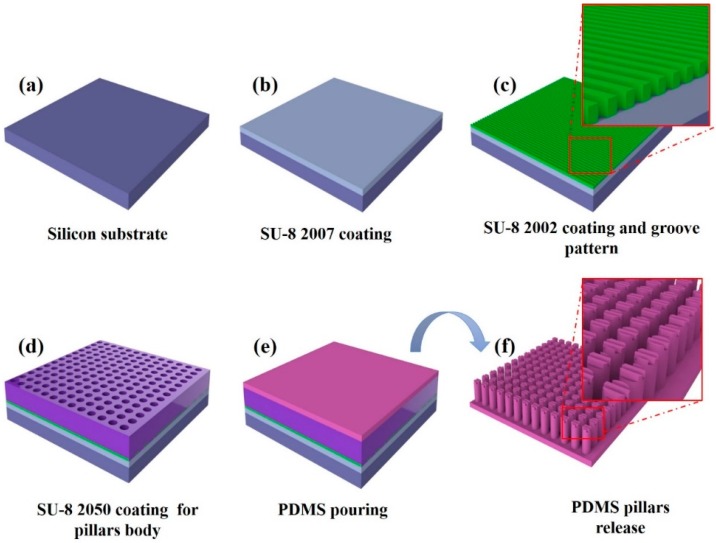
Process flow for the fabrication of PDMS pillar arrays with μgrooves. (**a**) 4 inch silicon substrate; (**b**) coating SU-8 2007 thin layer for increasing adhesion between the silicon substrate and SU-8 2002 layer; (**c**) coating SU-8 2002 negative photoresist for µgroove pattern; (**d**) coating high viscosity SU-8 2050 for µpuillar negative body mold; (**e**) PDMS pouring in to the negative mold; (**f**) PDMS layer release from the SU-8 mold.

The spring constant (k) of the designed µpillar is an important factor in the cardiomyocytes’ contraction force. Equation (1) shows the spring constant for each µpillar, based on the Young’s modulus of the PDMS and the µpillar’s geometry [31].

(1) $$k=\frac{3{\pi \text{ED}}^{4}}{64L^{3}}$$

where E is the Young’s modulus of the PDMS in the range of 0.5 to 4 MPa, depending on the fabrication process (including, mixing ratio, baking time, temperature, etc.) [32]. D and L are the diameter and length of the µpillar, respectively. A universal testing machine of strain and stress (Shimadzu, EZ-L, Kyoto, Japan) was used for accurate measurement of the mechanical properties of the PDMS during manufacturing the µpillar arrays. The measurement results showed that the PDMS was comparatively soft (0.5 MPa). Additionally, considering the designed structure of the µpillar, the finite element method (FEM) was used to predict the pillar’s displacement based on the applied force. The elemental analyzes of PDMS μpillar arrays are illustrated in Figure 2a. The designed µpillars showed good flexibility with a high aspect ratio. The displacements of μpillar arrays corresponding to applied forces were computed as shown in Figure 2b. It is seen that displacement of μpillar arrays is a function of applied force. From the simulation result, a spring constant of 0.04 N/m was observed for 16 μm diameter and 48 μm in length of µpillar. This calibration enabled accurate quantitative measurement with an elastomeric μpillar, and that is suitable for fabricating groove pattern on the top of μpillar. The stepwise fabrication process for the μpillar arrays with microgrooves (µgrooves) on the top of μpillar arrays is illustrated in Figure 3. A silicon wafer was used as a substrate (Figure 3a). A thin polymer layer made with a negative photoresist (PR, SU-8 2007) was spin-coated on the silicon wafer as shown in Figure 3b. The thin SU-8 layer enhances the adhesion force between the μgrooves and the silicon substrate. Next, SU-8 2007 was diluted with thinner at a ratio of 1:1. The diluted photoresist (PR) was spin-coated on the first SU-8 layer and then photo-lithographically patterned with an optical power of 60 mJ/cm^2^ using a conventional mask aligner. μgrooves with pitch of 2 µm and a depth of 0.5 µm were formed on the adhesion layer as shown in Figure 3c. A third layer was employed with thickness roughly of 48 μm as a permanent mold for pillar fabrication (Figure 3d) using different negative PR (SU-8 2050). For the fabrication of PDMS μpillars, base and curing agent were mixed in the ratio of 10:1. During the mixing process bubbles were generated in the PDMS solution that carefully removed in a vacuum dedicator for 30 min. Then, the PDMS was poured into the fabricated SU-8 mold, and bubbles were removed in a vacuum a second time. PDMS was hardened in the SU-8 mold at 70 °C for 4 h and cleaned with DI water (see Figure 3e). Finally, PDMS layer was carefully released from the mold as shown in Figure 3f. μpillar arrays without μgrooves were manufactured using the same (existing) method [21].

### 2.2. Cell and Culture Conditions 

All animal testing was performed after receiving approval from the Chonnam National University Animal Ethics Committee. The ventricles were harvested from one-day-old neonatal Sprague-Dawley rats. The ventricular tissue was digested in a mixture of 0.4 mg/mL collagenase and 0.6 mg/mL pancreatin to separate the cardiomyocytes from the tissues. After separating the cardiomyocytes and the fibroblast layer with centrifugal separation using Percoll, cardiomyocytes with high purity were gathered. The prepared cardiomyocytes were seeded at the top of µpillar arrays with 1000 cells per mm^2^ and cultured in an incubator at 37 °C with 5% carbon dioxide.

Factors such as the composition and formation of media, CO_2_ supply, culture temperature, etc., [33] influenced the cell culturing. These factors are controlled via the interaction between the cells and the substrates, the solubility, etc.; controlling these factors influences the cell growth. The culture medium was made from DMEM 67% (Dulbecco’s modified Eagle’s medium, LONZA, Seoul, Korea), M199 17% (heparin sodium salt from porcine intestinal mucosa, Sigma-Aldrich, Seoul, Korea), horse serum 10% (Sigma-Aldrich), fetal bovine serum (FBS) 5% (Sigma-Aldrich), and penicillin-streptomycin 1% (100× in stock, Sigma-Aldrich). In the culture medium, in addition to the carbon source, energy source, nitrogen source, inorganic salt, and trace elements, there is a buffering agent included. FBS includes an element for promoting cell growth and activity. Additionally, 1% penicillin-streptomycin was used as an antibiotic [34]. The culture medium was replaced every three days.

### 2.3. Immunocytochemistry 

Immunocytochemical staining was performed to identify the degree of alignment of cardiomyocyte grown on the pillars. First, the cardiomyocytes were fixed using a formalin solution (3.7%) at room temperature (RT) for 10 min and then washed with phosphate-buffered saline (PBS, Takara). Permeabilization was accomplished with 0.2% Triton-X (Sigma-Aldrich) in PBS for 15 min (RT). To prevent non-specific antibody binding, 1% bovine serum albumin in PBS (1% BSA, Sigma-Aldrich) was added and cultured for 40 min (RT). The primary anti-body [monoclonal anti actin (α-sarcomere)] was diluted as 1:200 with 1% BSA and incubated at RT for 1.5 h. The secondary antibody was (Alexa-Flour 488 goat anti-mouse IgG conjugate) diluted 1:500 in same blocking solution and incubated 1 h in RT. Finally, for nuclear staining, a DAPI solution (4,6-diamidino-2-phenylindole, Sigma-Aldrich) was added and cultured for 15 min at 37 °C.

### 2.4. Real-Time Recording of Cardiomyocyte and Data Analysing Methods 

After cell seeding, cardiomyocytes were cultured on the micro pillar array for three days after measurement was made. In pre-cultured time the cells were adhered to pillars, developed their myofibrils and contracted against the mico pillars. Micro pillars make a displacement due to the cardiomyocyte contraction and relaxation. Consequently, processes are recorded using an inverted microscope (ECLIPSE TS 100, Nikon, Tokyo, Japan) with a 25 fps (frame per second) sampling rate. From analysis of the videos, the deflection of a post, d, during a contraction was determined by Equation (2) [25],

(2) $$d=X_{i}-X_{ref}$$

where $X_{i}$ is the position of the pillar in the *i*th video frame during the contraction and $X_{ref}$ is the reference position taken at a point in time between contractions. The maximum instantaneous contraction force, F, is calculated using the spring constant of the micro pillar, k, shown in Equation (3).

(3) F=dk

The force a pillar produced during the relaxation state was controlled by differences in its relaxed position ($x_{ref}$) and original position. A GUI-based image analysis program developed to quantitatively evaluate the contraction force of cardiomyocytes with the help of MATLAB (Mathwork, Natick, MA, USA). The developed program allowed for quick and easy analysis of the µpillar arrays’ contraction force and alignment.

### 2.5. Statistical Analysis

Statistical analysis was performed using Student’s *t*-test to evaluate the statistical significance between the different groups. The data were presented as the mean ± standard error of the mean (SEM) for at least five independent experiments. The significance levels were set at * *p* < 0.05 and ** *p* < 0.01.

## 3. Results and Discussion 

### 3.1. Fabricated Micro Pillar Array Structure and Cell Growing Conditions

**Figure 4:**
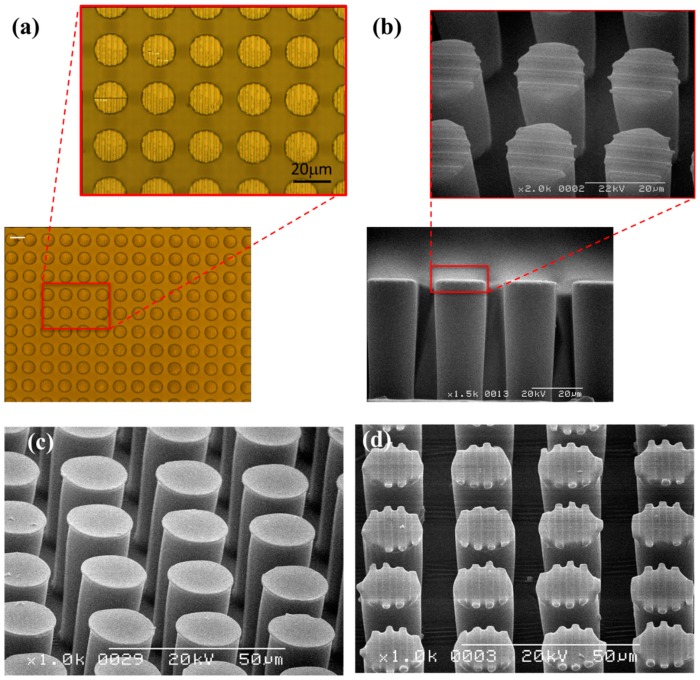
(**a**) Optical image of SU-8 negative mold (scale bar 20µm); (**b**) cross-sectional SEM view images of μgroove pattern on pillar arrays. 45°-tilted SEM micrographs of μpillar arrays (**c**) without and (**d**) with μgrooves.

Figure 4a shows optical microscope images of the top of µpillar arrays with µgrooves. The side and magnified top view micrographs of μpillar arrays with microgrooves were visualized using a scanning electron microscope (SEM) (Figure 4b). The manufactured µpillar arrays with µgrooves have a diameter and length nearly of 16 μm and 48 μm, respectively. The distance from the center of one pillar to the center of another pillar was roughly 23 μm. 45°-tilted SEM micrographs for μpillar arrays without and with μgrooves are shown in Figure 4c,d, respectively. The manufactured µgrooves have a line/space and a depth of 1.5 μm and 0.5 μm, respectively.

**Figure 5:**
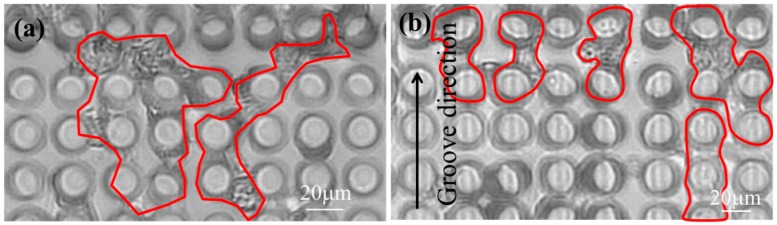
Top view of optical images of cardiomyocytes seeded on μpillar arrays (**a**) without and (**b**) with μgrooves (6-day).

NRVM (neonatal rat primary myocyte) was seeded onto the two different surface micro pillars. In the initial stage of NRVM seeding, the same quantity of cells was distributed uniformly on the micro functional surface. After 24 h of cell culturing, cardiac cells randomly oriented on the flat surface and those on the micro grooves were found oriented along the axis of micro groove. After cell pre-culturing (72 h), no significant difference was observed in distribution and spreading of cells. Accordingly, substantial contractile performance was observed at the same instant. Figure 5a,b shows the top view optical microscope images of cardiomyocytes seeded on μpillar arrays without and with μgrooves after 6-days, respectively. The direction and bending of the µpillars changed due to the cardiomyocytes’ contraction force. In the optical microscope images of µpillar arrays without µgrooves, the cardiomyocytes are connected anisotropically. Conversely, in µpillar arrays with µgrooves, the cardiomyocytes are connected isotropically along with the groove in direction. This result indicates that the cardiomyocytes grew along the direction of µgrooves formed on the tops of the µpillars. To more clearly show groove surface effects on cardiac cell growth function, the same local area was observed with the same cell number (*n* = 6).

**Figure 6:**
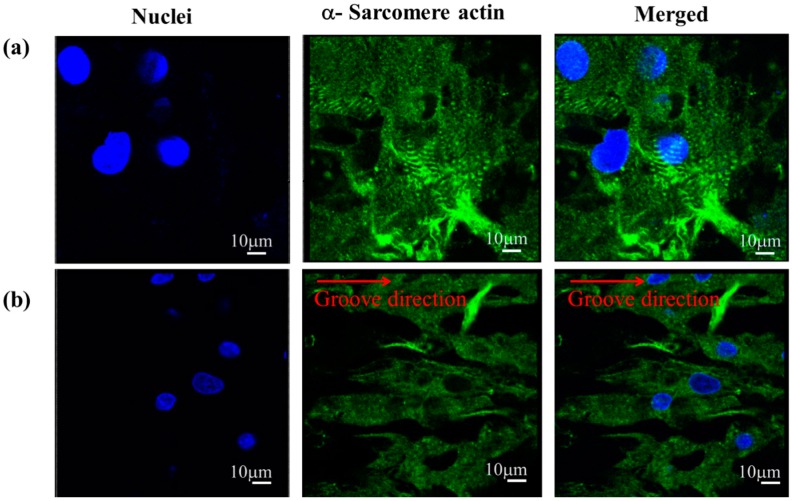
(**a**) Confocal images of immunofluorescence staining of cardiomyocytes on μpillar arrays row; (**a**) without and (**b**) with μgrooves. Images were indicated that; left columns as nuclei (blue), center column asα-sarcomere actin (green), and right column as merged images. The direction of μgrooves is indicated as a red line.

Figure 6a,b show the cardiomyocytes’ immucytochemisty staining images of cardiomyocytes on μpillar arrays without and with μgrooves, respectively. From the figures, it is seen that the cardiomyocyte nuclei are noted as blue color dots, while the actin filament is green in color. The immunocytochemictry staining images clearly suggest that the cardiomyocytes grew isotropically in case of μpillar arrays without μgrooves. However, μpillar arrays with μgrooves, the cardiomyocytes were arranged based on the direction of the µgrooves.

### 3.2. Measuring Contraction Force 

**Figure 7:**
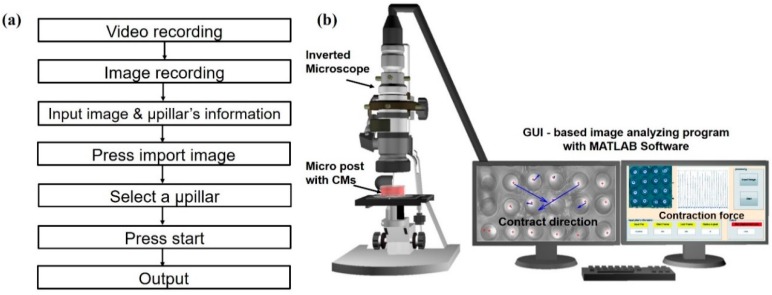
(**a**) Flow chart for the image analysis process; (**b**) vector analysis results for monitoring the alignment and contraction force of cardiomyocytes on the μpillar arrays.

The developed program allowed for quick and easy analysis of the µpillar arrays’ contraction force and alignment. Figure 7a shows the flow chart for the image analysis process. First, an inverted microscope (at 25 fps) was used to evaluate the mechanics and physiology of the cardiomyocytes cultured on the top of µpillar arrays. The preprocessing step was performed using ImageJ to show the top of the µpillars. After pre-processing, each frame was imported into the GUI-based image analysis program and to track the top of user-specified µpillars. The displacement data of µpillars were derived from the tracking information, which contains the change in position of a µpillars for each frame. With a data array, the pixel distance is converted into a corresponding deflection based on the pixel to micrometer conversion value. The quantitative analysis of the µpillar arrays is possible using MATLAB and corresponding contraction direction was computed as shown Figure 7b. This process was repeated for each µpillar and corresponding captured video. The resultant window displays the graph of displacement of the tracked µpillar arrays.

**Figure 8:**
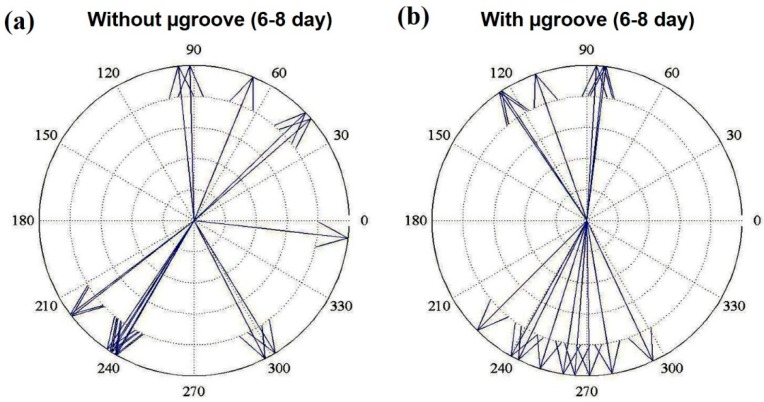
Vector analysis results of cardiomyocytes beating, recorded from day 6 to day 8 on μpillar arrays (**a**) without and (**b**) with μgrooves.

As mentioned earlier, the µpillar arrays’ displacement is an important factor for quantitatively evaluating the cardiomyocytes’ contraction force. The force of the pulsatile, regular contracting motion of cultured cardiomyocytes was estimated from the analysis of micropillar with maximum displacement determined by optical observation. To measure the cardiomyocytes’ contraction force, cardiomyocytes were grown on the top of the µpillar arrays. A custom MATLAB (Mathworks, Natick, MA, USA) program was used to determine the contraction direction of cardiomyocytes on the top of each µpillar. The optical image analysis programs are very useful for tracking and monitoring the deflection of individual μpillar or µpillar arrays. Further, the displacement vector of the cardiomyocyte contraction force is calculated using the deflections. Figure 8 presents the vector analysis results of μpillar arrays (Figure 8a) without and (Figure 8b) with μgrooves of the cardiomyocytes beating from 6-day to 8-day. The alignment of an individual cell along with the μgrooves was described by the angle between the major axis of the cell and μgrooves. As shown in Figure 8a, the arrangement of cardiomyocytes was isotopic, hence cell aggregates appear randomly. However, on the μpillar arrays with μgrooves, they repeatedly contracted and relaxed in directions centered on 90° and 270° as a seen in Figure 8b. The cardiomyocytes were influenced by the µgrooves such that they contracted in these directions. The experimental result shows that the proposed functional surface of μpillar array has potential for drug side-effect screening.

**Figure 9:**
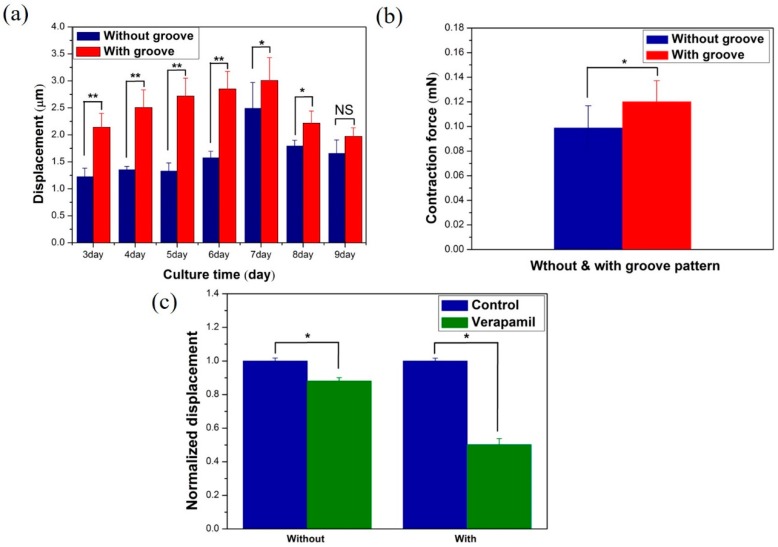
(**a**) Displacement of μpillar arrays without and with μgrooves from 3-day to 9-day (statistical significance difference between two column is denoted by asterisks * *p* < 0.05, ** *p* < 0.01 and NS-non significance); (**b**) Comparison of contraction force between μpillar arrays with and without μgrooves. (* *p* < 0.04, Student *t*-test) (**c**) Displacement change in μpillar arrays without and with μgrooves after Verapamil treatment at a concentration of 500 nM (* *p* < 0.04 and * *p* < 0.03 respectively, Student *t*-test).

The resulting data from the image analysis program confirmed that the cultured cardiomyocytes repeatedly contracted and relaxed in a uniform direction. If cardiomyocytes are attached to the top-sidewall of the pillar, then there is the possibility to produce large displacements. In the present work, it is observed that the cell position with 2-image planes (μpillar top plane and reference plane) and the polymeric groove environment is closely related to the cell organizational level as a tissue. That is the important part in cell growth on the surface, like a real tissue. After a 2-day stabilization phase, evaluation was recorded for a total of seven days, day 3 to day 9. The displacement generated in the µpillar arrays without and with µgrooves was statistically computed with the help of the bar diagram as shown in Figure 9a. Greater displacement was generated in the µpillar arrays with µgrooves than that in those without µgrooves because the µgrooves caused the cardiomyocytes to contract isotopically [24]. Also, actin cytoskeleton-dependent substrate geometry as seen in Figure 6a,b also plays a vital role in contraction and relaxation of cardiac muscle cell [30]. The displacement increases with culture time and observed maximum displacement of 3.0071 ± 0.42 μm on 7-day. Further, the pillar displacement decreases (after 7 days) with culture time. The present result shows the linear increase the contraction force at a certain range that indicates the force is maintained at an optimal value [16,25]. In other words, primary cell has finite life time spun depending on cell source type and growing function [30]. The highest displacement was observed on day 7 with 20% improvement in the µpillar arrays with grooves, compared to the µpillar arrays without grooves. Figure 9b shows the average displacement converted into a contraction force for µpillar arrays without and with µgrooves on the 7-day. The converted contraction force is 0.099 ± 0.017 μN and 0.1202 ± 0.016 μN (* = *p* < 0.04 student *t*-test) for µpillar arrays without and with µgrooves, respectively.

To evaluate the cardiomyocytes’ drug toxicity, Verapamil was diluted in ethanol to a concentration of ≤0.1%. Verapamil is a Ca^2+^ channel blocker, which decreases the contraction force of cardiomyocytes. The drug treatment was utilized on day 6 after the cell culture day (the contraction force maximized point). It is important to verify the drug toxicity’s influence on the cardiomyocytes’ contraction force, using a stock solution of Verapamil prepared in 100% ethanol and dimethyl sulfoxide (DMSO, Sigma-Aldrich). In the initial test results, no effect was observed on the cardiomyocytes’ contraction force or beating frequency for ethanol concentration of ≤0.1%. Figure 9c shows the change in normalized maximum displacement of μpillar arrays without and with μgrooves after Verapamil treatment at a concentration of 500 nM. The change in the cardiomyocytes’ contraction force caused by drug side effects was measured after drug treatment. The contraction force decreased to 12% ± 0.017% (Figure 9c without μgroove, * *p* < 0.04 Student *t*-test) for those without μgroove and 48.8% ± 0.016% for those with μgroove (Figure 9c with μgroove, * *p* < 0.03 Student *t*-test) 500 nM ethanol concentration. The remarkable decrease in contraction due to verapamil in the case of micro pillars with microgrooves means that the microwave surface pattern is more sensitive for assessing drug toxicity, in comparison with flat micro pillar arrays.

Comparing the present results with the previous study, the contraction force increases 2 times compared to that in research by Rodriguez et al. in μpillars without a groove pattern and is 2.5 times smaller than used SU-8 cantilever [16]. Also, it is strongly dependent on the applied cell number and spring constant of the sensor. In the case of cantilever sensor, they mainly focused on measuring the contraction force on the tissue level, not a single cell. The fabricated novel structure of micro pillar sensor’s essential feature was compared to the other contractile force measurement technique. The groove pattern is usually utilized on the flat surface (PDMS and SU-8 biocompatible materials) to provide continuous cell alignment to enhance contraction force [16,23,24,25]. However, in the present study, we have used a groove pattern on the separated individual micro pillar top side. It is also efficient for enhancing contraction force and gives us a chance to measure single cell mechanical performance at every local area with abnormal beating conditions. The fabrication process of the micro pillar is quite easy and utilized a simple double layer SU-8 negative micro molding technology to produce a three-dimensional surface micro pillar array. The main benefit of the present study, the cell force measurement system, provides a cell morphological environment with the grooved micro array that is very close to natural growth morphology, with similarity to cardiac muscle cells [35].

## 4. Conclusions

In this research, a micro-molding process was used on double-layered SU-8 structures to create PDMS µpillar arrays with µgrooves. The cultured µgrooves structures of µpillar arrays were used to induce cardiomyocyte growth along the direction of the µgrooves. The cardiomyocytes’ contraction force and direction were quantitatively evaluated using a GUI-based image analysis program. MATLAB was used to develop a graphic user interface program that allowed a rapid, simultaneous analysis of the deformation of a large number of µpillars. On the μpillar arrays with μgrooves, the cardiomyocytes repeatedly contracted and relaxed in the direction of the μgrooves; the contraction force was the greatest on day 7. The aligned cardiomyocyte contraction force was improved by a maximum of roughly 20%. Furthermore, the Ca^2+^ channel blocker Verapamil proves that mechanics and physiology analysis for drugs is possible. In the future, the proposed structure could be useful in a high-throughput drug screening system.
